# Peptides as epigenetic modulators: therapeutic implications

**DOI:** 10.1186/s13148-019-0700-7

**Published:** 2019-07-12

**Authors:** Yorick Janssens, Evelien Wynendaele, Wim Vanden Berghe, Bart De Spiegeleer

**Affiliations:** 1 Drug Quality and Registration (DruQuaR) group, Faculty of Pharmaceutical Sciences, Ghent University, Ottergemsesteenweg 460, 9000 Ghent, Belgium; 2 Protein Science, Proteomics and Epigenetic Signaling (PPES), Department Biomedical Sciences, University of Antwerp, Universiteitsplein 1, 2610 Wilrijk, Belgium

**Keywords:** Peptides, Epigenetics, Therapeutical development, Drug repositioning

## Abstract

Peptides originating from different sources (endogenous, food derived, environmental, and synthetic) are able to influence different aspects of epigenetic regulation. Endogenous short peptides, resulting from proteolytic cleavage of proteins or upon translation of non-annotated out of frame transcripts, can block DNA methylation and hereby regulate gene expression. Peptides entering the body by digestion of food-related proteins can modulate DNA methylation and/or histone acetylation while environmental peptides, synthesized by bacteria, fungi, and marine sponges, mainly inhibit histone deacetylation. In addition, synthetic peptides that reverse or inhibit different epigenetic modifications of both histones and the DNA can be developed as well. Next to these DNA and histone modifications, peptides can also influence the expression of non-coding RNAs such as lncRNAs and the maturation of miRNAs.

Seen the advantages over small molecules, the development of peptide therapeutics is an interesting approach to treat diseases with a strong epigenetic basis like cancer and Alzheimer’s disease. To date, only a limited number of drugs with a proven epigenetic mechanism of action have been approved by the FDA of which two (romidepsin and nesiritide) are peptides. A large knowledge gap concerning epigenetic effects of peptides is present, and this class of molecules deserves more attention in the development as epigenetic modulators. In addition, none of the currently approved peptide drugs are under investigation for their potential effects on epigenetics, hampering drug repositioning of these peptides to other indications with an epigenetic etiology.

## Background

The term epigenetics was first coined by Conrad Waddington in 1942 [1]. It refers to all heritable alterations in gene expression and chromatin structure due to biochemical modifications that do not change the primary gene nucleotide sequence [2]. These epigenetic tags are passed through to the offspring by epigenetic modifications in the germline cells [3]. However, this central dogma of non-genetic inheritance is under pressure as studies also suggest that sperm cells are epigenetically reprogrammed and reconstructed after fertilization [4]. The main mechanisms of epigenetics include DNA methylation, histone post-translational modifications (hPTMs) and variants, chromatin remodeling, and non-coding RNAs (ncRNAs). These epigenetic modifications are influenced by environmental and lifestyle factors such as diet, the microbiome, physical activity, and pollutants/toxins [5–7]. Moreover, the different epigenetic mechanisms can affect one another, adding extra layers of epigenetic regulation [8–10]. For instance, histone methylation can help directing DNA methylation patterns and DNA methylation can act as a template for histone modifications [8].

DNA methylations are the most common epigenetic modifications: a methyl group is transferred from S-adenosylmethionine (SAM) to the 5′ position of the cytosine ring by a DNA methyltransferase (DNMT) [11]. It is generally correlated with gene repression by direct blocking of transcription factor binding or by recruitment of chromatin remodeling readers [12, 13]. DNA methylation plays important roles in genomic imprinting, genome stability, and suppression of retrotransposons. Abnormal DNA methylation changes are associated with different forms of cancer and fragile X syndrome [12, 14, 15]. DNA methylation is a relatively stable process while demethylation, which is necessary for gene reprogramming, occurs via passive dilution or an active hydroxymethylation process [16, 17].

Histones associate with DNA to form nucleosome complexes. Nucleosome compaction of DNA protects the genome from DNA-damaging agents and allows to pack all DNA in the nucleus [18]. The best studied histone modifications are acetylation by histone acetyltransferase (HAT) enzymes and methylation by histone methyltransferases (HMT). Acetylation neutralizes the positive charge of lysine on the histones which results in chromatin unfolding and gene activation while methylation exerts its functions indirectly by recruitment of non-histone proteins of which the outcome (i.e., gene activation or repression) is dependent on the histone mono-di-tri-methylation motif, the specific combination of modifications (histone code) and histone localization along the genetic sequence [19, 20]. The reversed phenomenon is catalyzed by deacetylases (HDAC) and histone demethylases, respectively. To date, 18 different HDACs are identified and classified in four different classes. Other modifications such as butyrylation and succinylation exist [11]. All these modifications can alter gene expression by chromatin remodeling, but also play important roles in the DNA damage response [20]. In addition, canonical histone proteins can be replaced during cell cycle by non-canonical histone variants, which are encoded by separate genes. These variants can change the chromatin properties by destabilizing the nucleosome and altering the hPTM pattern which can influence gene transcription, DNA replication and repair, packaging, and segregation [21].

Next to the aforementioned DNA and histone modifiers, non-coding RNAs (ncRNAs) add another regulatory layer to the epigenetic machinery. The majority of these RNA molecules do not encode for proteins but can exert a variety of functions like regulating gene expression and DNA methylation or chromatin complexes [22–24]. Moreover, its regulatory functions are controlled by a plethora of epitranscriptomic modifications [25–30]. ncRNAs are classified based on their size: ncRNAs shorter than 200 nucleotides (nt) are called short non-coding RNAs (encompassing miRNA, siRNA, piRNA), whereas the ones larger than 200 nt are called long non-coding RNAs (lncRNAs) [31, 32]. The most known and most investigated group of ncRNAs is the microRNAs (miRNAs). These RNA molecules are about 19–24 nt long and repress gene expression by targeting mRNA. The miRNA recognizes 6–7 nucleotides in the 5′ untranslated region (“seed” region) of its target and guides an RNA-induced silencing complex (RISC) to the target mRNA. When the miRNA is completely complementary with its target, it acts as an endonuclease and degrades the mRNA. However, in the majority of the cases, miRNAs are only partially complementary and inhibit translation by relocation of the mRNA-miRNA-RISC complex to processing bodies where mRNA degradation occurs [33]. Since the seed region is relatively short, one miRNA is able to regulate hundreds of different genes [34]. miRNAs not only target mRNA, they can also regulate the expression of other ncRNAs adding an extra layer of epigenetic regulation [35]. To date, more than 1800 putative miRNAs are identified and collected in the miRBase database [36]. siRNAs have a similar mechanism of action but differ from miRNA by source of origin and have subtle structural differences; miRNA consists of an incomplete hairpin-shaped double-stranded RNA which is processed by Drosha and Dicer, whereas siRNA is the product of fully complementary double-stranded RNA which is only processed by Dicer [32]. lncRNAs can act as precursors for short ncRNAs or regulate gene expression at different levels, mainly by chromatin remodeling [37]. More recently, it has been found that these lncRNAs can also have, despite their name, a coding function for small peptides [38, 39].

In this review, we discuss the influence of peptides on different regulatory layers of epigenetics. In addition, we give an overview of already approved drugs with epigenetic effects and discuss the potential of therapeutic peptides in this field.

## Epigenetics and disease

Dysregulated epigenetics is involved in a wide range of diseases. The reversible nature of epigenetic modifications makes them interesting therapeutic targets [11]. Epigenetic control can already be dysfunctional during embryonic development, possibly caused by increased oxidative stress in sperm cells, and result in congenital diseases such as fragile X syndrome and Hirschsprung disease [15, 40–42]. Later in life, adverse epigenetic regulation can result in a variety of diseases like cancer [43], blood disorders [44], neurological and neurodegenerative disorders [45–48], and respiratory disorders [49, 50]. These epigenetic shifts can be used as diagnostic markers in both invasive and non-invasive samples [51–54].

## The influence of peptides on epigenetic mechanisms

### Endogenous peptides

**Fig. 1:**
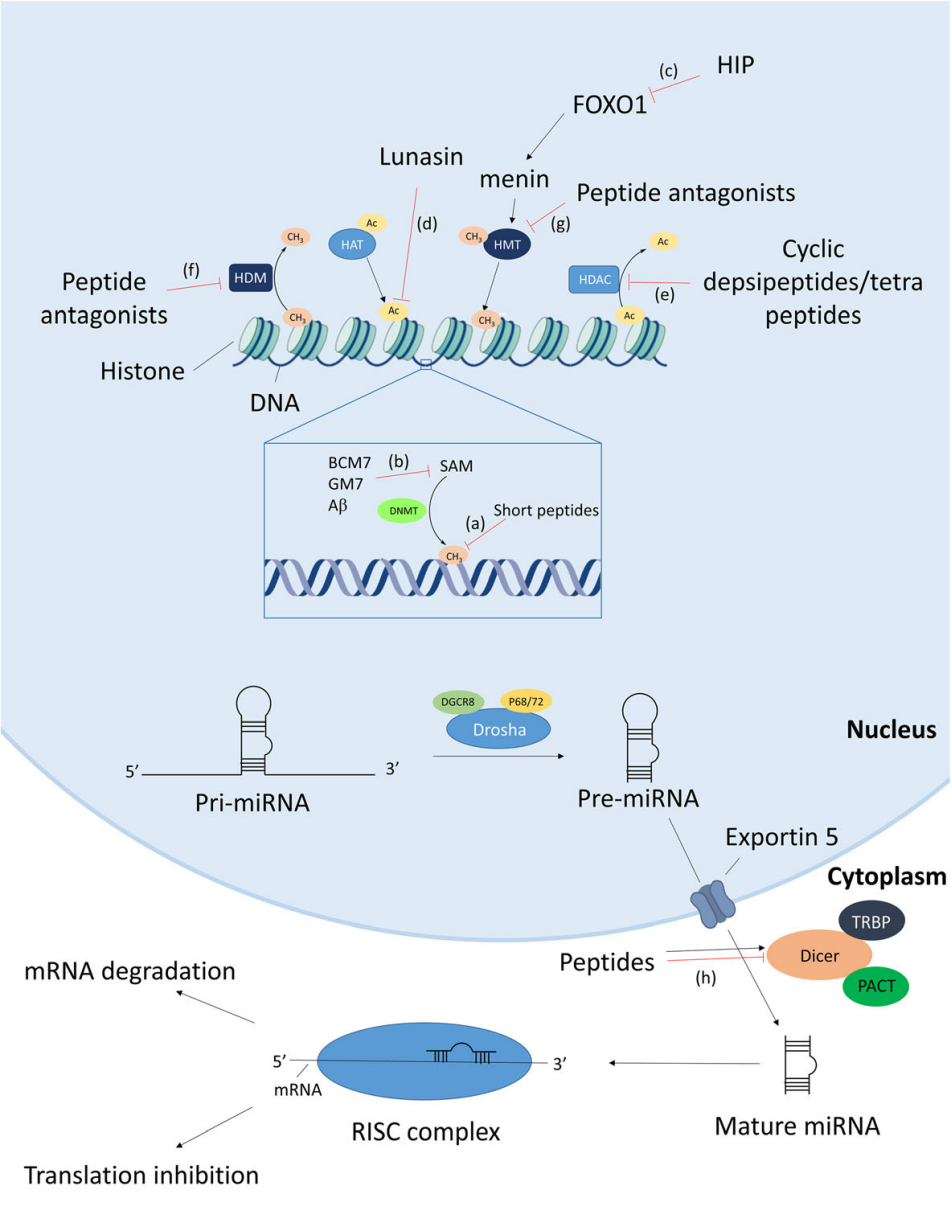
Schematic overview of peptide effects on different epigenetic mechanisms. (a) Short peptides inhibit DNA methylation by blocking DNA methyltransferase binding or initiation of strand separation. (b) BCM7, GM7, and Aβ suppress DNA methylation by inhibiting cysteine uptake and lowering the SAM/SAH ratio in the cell. (c) HIP inhibits histone methylation indirectly by suppressing the FOXO1 transcription factor and subsequent menin binding and histone methyltransferase recruitment. (d) Lunasin blocks H3 and H4 histone acetylations by preventing HAT interaction. (e) Cyclic depsi- and tetrapeptides inhibit histone deacetylases by interaction with zinc atoms in the binding pocket of the enzyme. (f, g) Synthetic peptide antagonists block the interaction sites of the HDM and HMT enzymes. (h) Synthetic peptides either promote or suppress Dicer-mediated maturation of miRNA

Peptides are small proteins buildup of less than 50 amino acids. These compounds exert a variety of functions in the human body and are able to modulate epigenetic mechanisms. There are different reports discussing gene regulatory effects of peptides [55–58]. Depending on the peptide, these effects are direct or indirect by binding to receptors and activation of intracellular signaling cascades (Table 1). Khavinson et al. discovered a variety of di-, tri-, and tetra-peptides in the nucleus. These short peptides directly interact with DNA in the promotor gene region, causing strand separation and initiation of gene transcription. If this is the case, then these short peptides of four amino acids and less can be considered as a separate class of epigenetic regulators. Another proposed mechanism of action is the binding of these peptides to the gene promotor region making it inaccessible for DNA methyltransferases, resulting in unmethylated promotor regions and gene activation [59, 60]. This way, the short peptides act as DNA methylation inhibitors (Fig. 1 (a)). These short “cryptic” peptides can be formed endogenously by proteolytic cleavage of nuclear proteins or by synthesis after which they penetrate the cytoplasmic and nuclear membrane [60, 61]. These peptides are involved in the epigenetic regulation of aging and can have health-promoting effects by for example suppressing age-related increased expression of matrix metalloproteases and caspase-dependent apoptosis [55, 93].

**Table 1:** An overview of the effects of peptides on different epigenetic systems

Epigenetic mechanism	Peptide	Effect	Type	Source	Status	Reference
DNA methylation	Short peptides	Inhibition	Direct	Endogenous/synthetic	Pre-clinical	[59–61]
	Aβ	Inhibition	Indirect	Endogenous	Pre-clinical	[62–64]
	BCM7	Inhibition	Indirect	Milk	Pre-clinical	[65–67]
	GM7	Inhibition	Indirect	Wheat	Pre-clinical	[66]
Histone methylation	HIP	Inhibition	Indirect	Endogenous	Clinical	[68]
	EZH2 antagonists	Inhibition	Direct	Synthetic	Pre-clinical	[69]
	WHSC1 antagonist	Inhibition	Direct	Synthetic	Pre-clinical	[70]
Histone demethylation	LSD1 antagonists	Inhibition	Direct	Synthetic	Pre-clinical	[71]
Histone acetylation	Lunasin	Inhibition	Direct	Soybean	Clinical	[72]
Histone deacetylation	Romidepsin	Inhibition	Direct	Bacterial	Approved	[73]
	Burkholdacs	Inhibition	Direct	Bacterial	Pre-clinical	[74]
	Spiruchostatins	Inhibition	Direct	Bacterial	Pre-clinical	[75]
	Thailandepsin	Inhibition	Direct	Bacterial	Pre-clinical	[76]
	FR901375	Inhibition	Direct	Bacterial	Pre-clinical	[77]
	Largazole	Inhibition	Direct	Bacterial	Pre-clinical	[78]
	Plitidepsin	Inhibition	Direct	Tunica	Clinical	[79]
	Chlamydocin	Inhibition	Direct	Fungal	Pre-clinical	[80]
	Trapoxins	Inhibition	Direct	Bacterial	Pre-clinical	[81]
	CHAP	Inhibition	Direct	Synthetic	Pre-clinical	[82, 83]
	Apicidin	Inhibition	Direct	Bacterial	Pre-clinical	[84]
	Microsporins	Inhibition	Direct	Fungal	Pre-clinical	[85]
	Azumamides	Inhibition	Direct	Sponge	Pre-clinical	[86]
	FR235222	Inhibition	Direct	Fungal	Pre-clinical	[87]
	AS1387392	Inhibition	Direct	Fungal	Pre-clinical	[88]
miRNA	LK-L1C/K6W/L8C	miR-29b ↑	Direct	Synthetic	Pre-clinical	[89]
	LKKLLKLLKKWLKLKGX LKKLLKLLKKLWKLKGX	miR-155 ↓	Direct	Synthetic	Pre-clinical	[90]
	L50	miR-21 ↓	Direct	Synthetic	Pre-clinical	[91]
LncRNA	BNP (Nesiritide)	LSINCT5 ↑	Direct	Endogenous	Approved	[92]

Amyloid-beta (Aβ), a 37–43-amino acid-long peptide which is the core component of senile plaques in Alzheimer’s disease (AD) reduces global DNA methylation but increases DNA methylation in the promotor region of the neprilysin gene, an enzyme responsible for Aβ degradation, hence downregulating its own production [62]. Soluble oligomers of Aβ are also able to decrease cysteine uptake in human neuronal cells causing a decrease in intracellular glutathione levels, accompanied by a global decrease in DNA methylation which may contribute to the AD pathology (Fig. 1 (b)) [63]. In Down syndrome patients, increased levels of plasma Aβ and a decreased methylation of three CpGs which have been reported to predict aging in adults are observed; these results indicate that Aβ contributes to the observed accelerated aging in Down syndrome patients [64]. Endogenous peptides are thus able to regulate gene expression and exert their effects by selectively increasing or decreasing DNA methylation.

The effects of endogenous peptides are not limited to DNA methylation but also include effects on histone modifications. The human pro-islet peptide (HIP), a 14-amino acid-containing peptide which is suggested as a therapeutic peptide for the treatment of diabetes, increases β-cell mass, and improves glycemic control. This peptide promotes differentiation of human fetus-derived pancreatic progenitor cells by promoting expression of different important pro-islet transcription factors through phosphorylation and inhibition of the FOXO1 transcription factor. This inhibition leads to a reduced menin binding to the promotor region of the pro-islet transcription factors and a subsequent decreased recruitment of H3K9 methyltransferases (Fig. 1 (c)). Thus, the HIP peptide exerts its effects by an indirect repressive effect on histone methylation in the promotor region of pro-islet transcription factors resulting in a promoted expression [68].

Peptides also affect the expression of ncRNAs. B-type-natriuretic peptide (BNP), a cardiac hormone secreted from the atrial and ventricular myocardium, promotes myocardial cell apoptosis during myocardial ischemia-reperfusion injury by upregulation of the lncRNA LSINCT5. This lncRNA regulates myocardial cell apoptosis via activation of the caspase-1/IL-1β pathway causing chronic heart failure. Thus, elevated BNP levels can result in chronic heart failure and is therefore an excellent diagnostic marker [92, 94]. These effects could explain the increased mortality risk after nesiritide treatment [95]. Nesiritide, a recombinant form of the peptide, is already been approved by the FDA since 2001 for the symptomatic treatment of acute decompensated heart failure due to its vasodilating activity. This product is not approved in the European Union and Japan. In the years following approval, its benefits were being questioned, and in 2011, it was demonstrated in a large-scale clinical trial (*n* = 7141) that nesiritide shows no benefits over placebo-treated patients and it was concluded that this medicine could not be generally recommended for routine use [96, 97]. Despite this information, the product is still not withdrawn from the US market.

### Food-derived peptides

Trivedi et al. demonstrated that beta-casomorphin-7 (BCM7) and GM7, cryptic peptides released by hydrolytic digestion of respectively casein and gliadin, decrease cysteine uptake in neuronal and gastrointestinal cells via activation of opioid receptors. This decrease is accompanied by increased oxidation of intracellular glutathione and increased DNA methylation at positions +65 to +80 of the gene transcription start sites resulting in downregulation of several genes of the transsulfuration pathway and methionine cycle [65, 66]. These results suggest that milk- and wheat-derived peptides exert antioxidant effects important during postnatal development by epigenetic mechanisms. BCM7 also promotes neurogenesis of neuronal stem cells by decreasing global DNA methylation (Fig. 1 (b)) [67].

Lunasin, a 43-amino acid soybean-derived polypeptide, is able to inhibit core histone acetylation of H3 and H4 and has been shown to exhibit marked anti-cancer activities (Fig. 1 (d)) [72, 98]. At its carboxyl-terminal end, the peptide contains 8 negatively charged Asp residues which act as the inhibitor of the positively charged H3 and H4 acetylations. This sequence is immediately preceded by an Arg-Gly-Asp (RGD) motif which is responsible for attachment to the extracellular matrix and facilitating cell penetration of the peptide, and a 9-amino acid-long α-helical structure which guides and binds lunasin to the core histone proteins [99]. Recently, it has been found that this peptide also exerts beneficial effects in neurodegenerative diseases such as AD and ALS [100, 101].

Next to modulating DNA methylation and histone modifications, food-derived peptides can also exert regulatory effects on ncRNA transcription. A peptide hydrolysate extract, i.e., a peptide mixture derived from the soft-shelled turtle (a functional food in Chinese traditional medicine), modulates the expression of 101 different miRNAs in human gastric cancer cells. Many of the upregulated miRNAs have tumor suppressive actions (they target oncogenes), making the peptide a potential therapeutic anti-cancer peptide [102].

The effects of food-derived peptides on epigenetics and their possible use in the treatment of diseases make these products subject of the discussion whether these should be considered as functional foods or as medicinal products (so-called borderline products). A medicinal product is seen as a product presented as having properties for treating or preventing disease in human beings or having restoring, correcting, or modifying physiological functions, while no single legislative definition currently exists for functional foods. Generally, it is stated that a functional food contains next to its nutritional impact also beneficial health effects [103]. In the case of “borderline products,” the European Court of Justice (ECJ) decisions are constructive: e.g., in the Hecht-Pharma case (Case C-140/07), the ECJ decided that a product composed of fermented red rice, which contains monacolin (a cholesterol-lowering molecule), should be considered as a food supplement, contrary to the German administrative authorities which classified it as a medicinal product. This decision was based on the fact that all the characteristics of a product are relevant for its classification. So whether a product with epigenetic bioactive peptides is classified as a medicinal product or as a food product depends on characteristics such as composition, pharmacological effect, manner of use, dosage, distribution, and familiarity of the risks to the consumers. Based on this ECJ ruling, pharmaceutical law only applies to products sold for treatment, cure, or prevention of human diseases and to products which, by composition, are scientifically proven to modify physiological functions [104].

### Environmental peptides

**Fig. 2:**
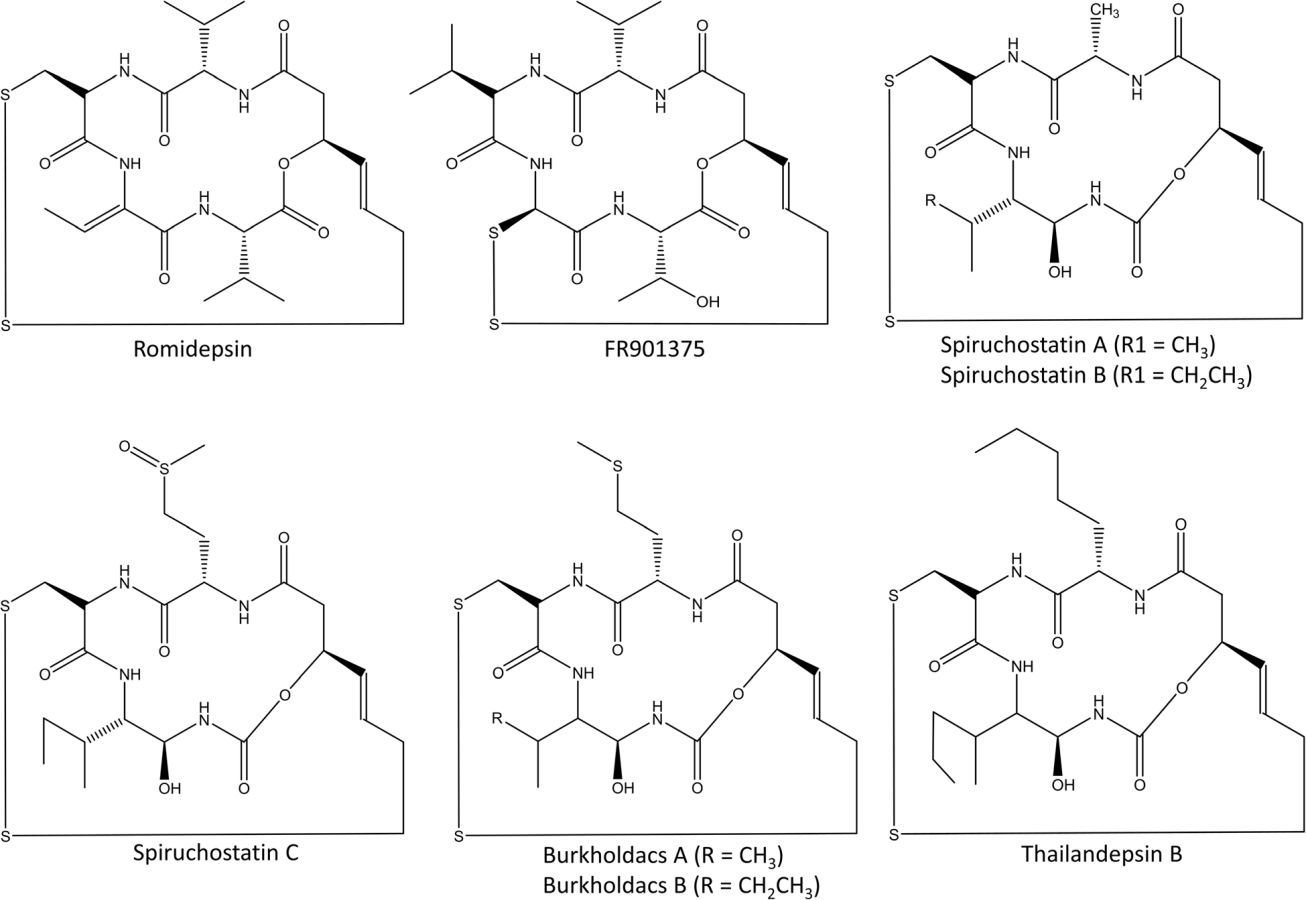
Structures of acyldepsipeptides

These peptides can be found in the environment and are mainly produced by microbial species. Romidepsin, a fermentation product of *Chromobacterium violaceum*, is the first FDA-approved peptide-based drug with epigenetic effects. It is a broad-spectrum HDAC inhibitor (HDACi) but is mainly active against class I HDACs. Within the cell, the disulfide bond of the peptide is reduced releasing thiol in this process. This thiol interacts with zinc atoms in the binding pocket of zinc-dependent HDAC, thereby inhibiting its activity (Fig. 1 (e)) [73]. Other depsipeptides, such as spiruchostatins (A, B and C), FR901375, largazole, plitidepsin, burkholdacs (A and B), and thailandepsin B, belong to the same group as romidepsin and have similar working mechanisms (Fig. 2) [74–76, 105]. The spiruchostatins, burkholdacs, and thailandepsin B all originate from the bacterium *Burkholderia thailandensis*. Burkholdacs compounds differ from the spiruchostatins by the substitution of methionine with alanine [74]. FR901375 and largazole are structurally closely related to romidepsin and are respectively fermentation products of *Pseudomonas chlororaphis* and the marine cyanobacterium *Symploca sp.* [77, 78]. Plitidepsin (Aplidin®) is a cyclic depsipeptide originating from the marine tunica *Aplidium albicans.* It has pleiotropic effects on cancer cells by binding to the eEF1A2 protein, which results in cell-cycle arrest, growth inhibition, and induction of apoptosis by different pathways and is also considered as a HDAC inhibitor [79, 106]. Numerous clinical trials have been conducted on the use of this peptide alone or in combination with other anticancer agents in the treatment of various cancer types. The most promising effect was the combined treatment in relapsed/refractory multiple myeloma together with dexamethasone [79, 107, 108]. However, the European Medicines Agency (EMA) refused market authorization of this product in 2018 based on the modest improvement in overall survival and the more frequent severe side effects that occurred. The EMA concluded that the benefits did not outweigh its risks for the proposed broad indications [109].

**Fig. 3:**
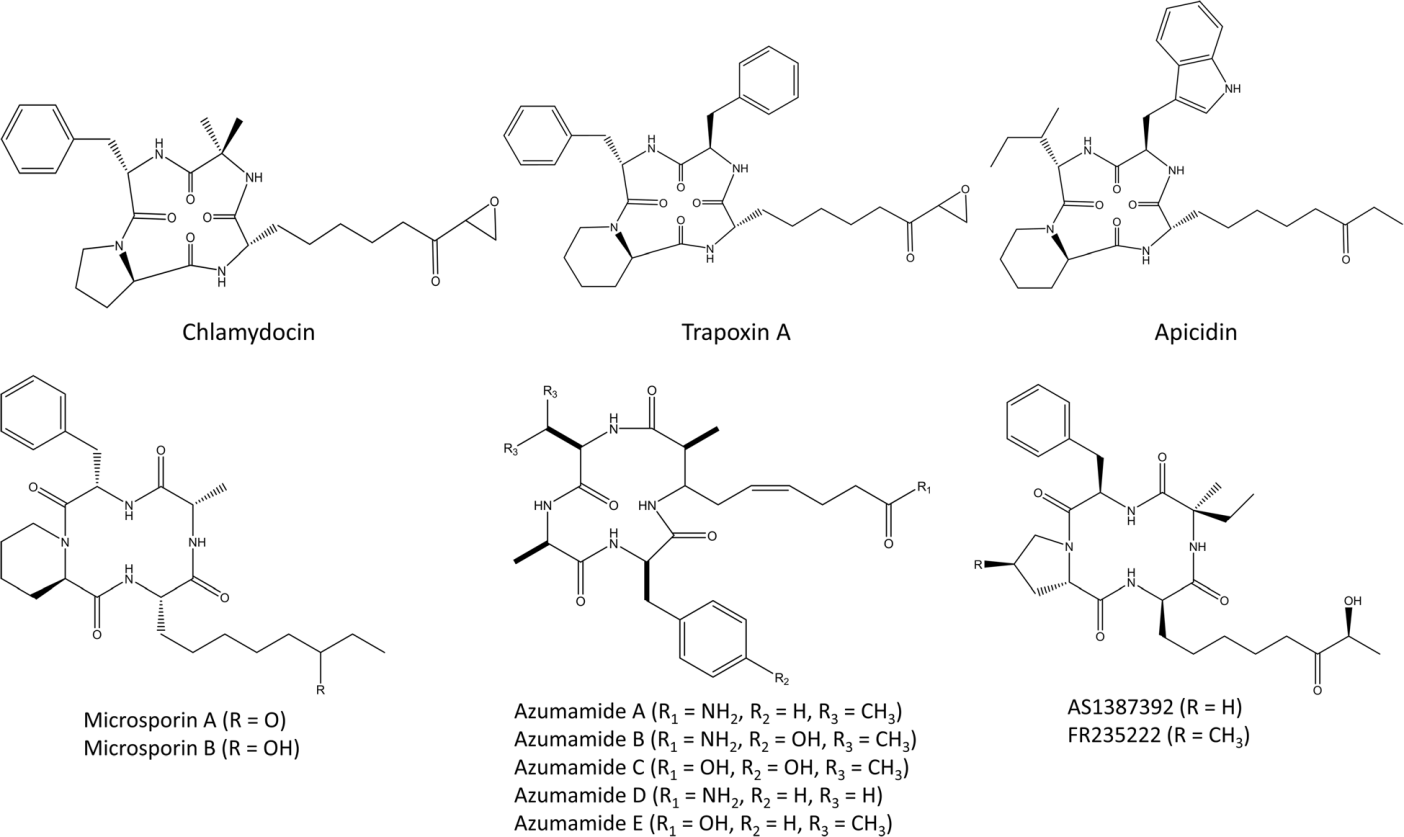
Structures of cyclic tetrapeptides

A second class of peptide HDACi are the cyclic tetrapeptides. Belonging to this group are chlamydocin, apicidin, FR235222, microsporins (A and B), azumamides (A-E), and the trapoxins (Fig. 3) [110]. Chlamydocin, a fungal metabolite with a strong HDAC inhibitory potency, induces hyperacetylation of histones H3 and H4, resulting in G2/M cell cycle arrest and induction of apoptosis by activating caspase-3. In addition, it downregulates survivin, an inhibitor of apoptosis which is selectively expressed in tumors [80]. Over the years, numerous chlamydocin analogues have been found or developed [110–113]. Trapoxin A is isolated from the fungal parasite *Helicoma ambiens*; just like chlamydocin, it contains a 6-oxo-7,8-epoxyoctyl side chain which serves as the Zn^2+^-coordinating group and its ketone carbonyl group is isosteric with the scissile carbonyl of the HDAC substrate acetyl-L-lysine, acting like an irreversible class I HDACi [81]. The trapoxin A and trichostatin A molecules are often combined replacing the epoxyketone structure of trapoxin A with a hydroxamic acid from trichostatin A resulting in a hybrid cyclic hydroxamic-acid-containing peptide (CHAP). This way, target enzyme specificity is affected which can lead to the development of isoform-specific HDAC inhibitors [82, 83]. Apicidin is structurally analogous to trapoxin A; it lacks the epoxyketon functional group but also shows HDAC inhibitory activity and has similar effects as chlamydocin [81, 84]. Microsporins A and B are isolated from the marine-derived fungus *Microsporum gypseum* and are together with azumamide A-E, derived from the sponge *Mycale izuensis*, the first marine-isolated cyclic tetrapeptides with inhibitory activities against HDAC [85, 86]. FR235222 and its analogue AS1387392 are two fungal metabolites, isolated from *Acremonium* species, that show immunosuppressive activities. These two cyclic tetrapeptides are able to inhibit T cell proliferation and lymphokine production by inhibiting histone deacetylase [87, 88]. The structure of these natural occurring cyclic peptides has been used several times to design more selective and potent peptide-based HDAC inhibitors [114, 115].

### Synthetic peptides

Lysine-specific demethylase 1 (LSD1) is a chromatin-remodeling enzyme which removes methyl groups from lysine at position 4 of histone H3. This enzyme plays an important role in cancer as over-expression results in silencing of tumor-suppressing genes, and it is thus regarded as an attractive target for therapeutic interventions. Different peptide-based inhibitors have already been developed; these peptides are analogues of the histone H3 lysine 4 substrate region of LSD1 and act as antagonists (Fig. 1 (f)). However, neither one is currently yet under human clinical investigation [71]. The structures of these peptide antagonists are modified to optimize the potency of these inhibitors. Forneris et al. created a peptide where lysine at position 4 is replaced by methionine, which resulted in an improvement in K_i_ from 1.8 to 0.04 μM [116]. Replacement of alanine at position 1 with a serine residue also resulted unexpectedly in a potent inhibitor [117]. Next to substituting amino acids, lysine can also be modified by more complex moieties such as trans-2-phenylcyclopropylamine or by cyclization [71, 118]. These modifications resulted in the development of inhibitors with IC_50_ values in the nanomolar range [119]. Another approach is to design peptide substrates which block the interaction of LSD1 with its target transcription factors, resulting in a more selective activation of LSD1 target genes [120].

EZH2 is a histone methyltransferase that catalyzes di- and trimethylation of lysine at position 27 of histone H3 and is linked to gene repression. Overexpression of this enzyme has been correlated to various cancer types due to epigenetic silencing of important tumor suppressor genes [121]. Smadbeck et al. used an in silico approach to design inhibitory peptides of EZH2. Using an integer linear optimization model, 17 sequences were predicted to have significantly higher binding affinities to EZH2 than the native H3-derived peptide (Fig. 1 (g)). From these 17 peptides, 10 peptides were selected for experimental validation using a HMT enzymatic assay, which assessed the EZH2-dependent transfer of tritiated methyl-groups from the SAM donor to reconstituted oligonucleosomes. This approach resulted in peptide inhibitors with IC_50_ values in the micromolar range [69]. However, this observed potency is significantly lower than those of previously discovered small molecule inhibitors of EZH2 [122]. Morrison et al. developed different HMT inhibiting norleucine containing peptides with IC_50_ values in the micromolar range. These peptides inhibit HMT from the NSD family and are derived from the histone H4 sequence [70].

Next to affecting DNA- and histone modifications, synthetic peptides can also interact with the hairpin structure of miRNAs, affecting their activity such as changing the efficiency of miRNA maturation. Inhibiting miRNA maturation results in suppressed miRNA formation and successive upregulation of the miRNA target genes. However, stabilizing miRNA maturation can result in an enhanced downregulation of the miRNA’s targets. This strategy can be used to target suppressors of p53, the main tumor suppressor protein, in new approaches for treating cancer. LK-L1C/K6W/L8C is an amphiphilic peptide that binds to the terminal loop region of pre-miR29b which matures to miR29b and induces apoptosis of cancer cells through p53 stabilization. Binding of this peptide to pre-miR29b improves the complexation with Dicer, the enzyme responsible for miRNA maturation, and enhances miR29b expression. This peptide is thus able to increase apoptosis induction in cancer cells by upregulation of miR29b and p53 [89]. MiR-155 is one of the most potent miRNAs that suppress apoptosis in human cancer cells and is overexpressed in numerous types of cancer. Using peptide microarrays, Pai et al. found two peptides which are able to inhibit Dicer-mediated miRNA-155 maturation, hereby upregulating the target genes of miRNA-155 and promoting apoptosis by caspase-dependent pathways. These peptide inhibitors bind to the apical stem-loop region of pre-miRNA, hereby blocking the interaction site of Dicer and suppressing the Dicer-mediated processing and are thus potential new therapeutics in treating various types of cancer [90]. Shortridge et al. discovered a macrocylic peptide which is able to bind pre-miR21 also hampering Dicer-mediated maturation to miR21, a miRNA overexpressed in many cancers, by screening an existing peptide library of 54 peptides [91]. Pre-miRNA interacting peptides that inhibit maturation can also be discovered using the phage display technology [123, 124]. These studies indicate that peptides can either promote or inhibit the Dicer-mediated maturation of pre-miRNA to mature miRNA, hereby upregulating important tumor suppressor genes or downregulating oncogenes which are overly expressed in cancer (Fig. 1 (h)). Targeting these epigenetic mechanisms, peptides or peptide analogues used as antagonists can have great potential to be used as epigenetic drugs for treating not only cancer, but a wide variety of diseases.

## Effects of epigenetics on peptide expression

Peptides can thus have an effect on multiple aspects of epigenetic regulation. However, endogenous peptide expression is also regulated by epigenetic mechanisms. For example, during withdrawal of both tobacco smoking and alcohol consumption, expression of natriuretic peptides and vasopressin are regulated by changing DNA methylation patterns in the promotor regions of the peptides [125–128]. HDACs are able to downregulate the expression of cationic antimicrobial peptides; therefore, treatment with HDACi can be an alternative approach to combat bacterial infections and tackle the problem of emerging antibiotic resistance by upregulating the expression of these antimicrobial peptides [129]. Also in cancer tissue, epigenetic mechanisms are responsible for the expression of cancer-specific peptides such as the trefoil factor family, consisting of peptides with pivotal roles in oncogenic transformation [130, 131]. Recently, cryptic long terminal repeat (LTR) transcription of endogenous retroviruses (ERV) has been observed as a novel class of treatment-induced non-annotated *out of frame* dsRNA transcripts (TINATs) upon epigenetic therapy with DNMT and HDAC inhibitors [132]. The resulting transcripts frequently encode truncated or chimeric open reading frames translated into products with predicted abnormal or immunogenic functions. While these out-of-frame transcripts are likely subjected to nonsense-mediated decay (NMD), chimeric peptide sequences encoded in TINAT fusion transcripts are potentially immunogenic based on their foreign sequence and their capability of being presented on MHC class I molecules for recognition by cytolytic T cells [132–134].

## Therapeutic implications

### FDA-approved epigenetic drugs

Since the strong involvement of epigenetic mechanisms in different diseases, molecules which inhibit or enhance these epigenetic modifications are being developed [51]. Prachayasittikul et al. have nicely summarized the currently available DNA methyltransferase inhibitors (DNMTi), histone methyltransferase- and demethylase inhibitors (HMTi and HDMi), histone acetyltransferase and deacetylase inhibitors (HATi and HDACi), sirtuin inhibitors (SIRTi), and bromodomain inhibitors (BRDi) [11]. Epigenetic drugs which are under investigation are also collected in a database named the “Human epigenetic drug database (HEDD)” [106]. To date, 11 drugs with epigenetic effects are approved by the FDA of which 7 are targeting different kinds of cancer by upregulating tumor suppressor genes (Table 2) [11, 135]. These drugs all target hematopoietic cancers as these cancer types are more sensitive to this type of drugs. However, combination treatment of solid tumors with epigenetic drugs and DNA damaging chemotherapeutics or immunotherapy can act in a synergistic way resulting in a more efficient treatment [135–137]. In 2009, the first peptide-derived epigenetic drug (romidepsin) was approved for the treatment of cutaneous T cell lymphomas (CTCL), and in 2011 for peripheral T cell lymphomas (PTCL) [73]. In addition, it has a potential use in other kinds of cancer, lung fibrosis, and Epstein-Barr infections [138–140]. Currently, it is the only FDA-approved peptide medicine with epigenetic effects together with nesiritide. However, nesiritide was, just like other traditional drugs (valproic acid, procainamide, hydralazine), not approved for its epigenetic effects but for their non-epigenetic medicinal effects. Their epigenetic properties were only discovered more recently, making them excellent candidates for drug repurposing [141–143]. Despite the FDA approval status of both romidepsin and nesiritide, the peptides are not approved in the European Union by the EMA [144, 145]. The EMA refused market authorization of romidepsin based on the absence of a treatment control group in the main clinical trial [146]. Furthermore, they did not authorize nesiritide as they are awaiting more long-term data on (renal) side effects and mortality of this product [145]. Recent evidence shows that valproic acid and procainamide can be efficacious in the treatment of cancer, whereas the first clinical trials using valproic acid as a combination therapy in advanced sarcomas have already started [142, 147]. Hydralazine has the potential to be used against chronic heart and kidney failure [143]. Pargyline, a monoamine oxidase inhibitor which was brought on the market in the 1960s as an antihypertensive drug, shows some sequence and structural similarity to demethylases from the LSD1/KDM1 family, and indeed, this compound is able to inhibit the histone demethylase KDM1A [148]. However, in 2007, this compound was discontinued and is no longer on the market. In 2014, an orally active HDACi (Chidamide) was approved in China for the treatment of recurrent and refractory peripheral T cell lymphoma; however, it is still not approved worldwide [149]. Several other molecules are currently in different phases of clinical trial [51].

**Table 2:** FDA-approved drugs with epigenetic effects

Drugs	Classification	Indicated disease	Approved year
Procainamide	DNMTi	Cardiac arrhythmia	1950
Hydralazine	DNMTi	Hypertension	1953
Valproic acid	HDACi	Epilepsy/seizures	1978
Nesiritide	LncRNA upregulator	Heart failure	2001
Azacitidine	DNMTi	MDS	2004
Vorinostat	HDACi	CTCL	2006
Decitabine	DNMTi	MDS	2006
Romidepsin	HDACi	CTCL/PTCL	2009/2011
Ruxolitinib	JAK1/2 inhibitor	Myelofibrosis	2011
Belinostat	HDACi	PTCL	2015
Panobinostat	HDACi	MM	2015

*MDS* myelodysplastic syndrome, *CTCL* cutaneous T cell lymphoma, *PTCL* peripheral T cell lymphoma, *MM* multiple myeloma

### Pharmaceutical peptide development for epigenetic therapy

Peptides represent a unique class of pharmaceutical compounds, residing in the gray area between small molecules and proteins yet therapeutically and biochemically different from both. The first use of peptides as therapeutics started in the 1920s, where insulin was used as a replacement therapy for treating diabetes. Peptides show several advantages over small molecules: they are highly selective and potent, are well tolerated (reduced toxicity), and have a predictable metabolization. In addition, peptide therapeutics generally have lower attrition rates during clinical development and a shorter time to market approval [96]. The peptide therapeutic market is an ever increasing market with an estimated annual growth rate of 9% between 2016 and 2024. Estimations indicate that this market will reach 50 billion dollars by 2024, thus becoming more and more important in the pharmacological landscape [150]. Techniques such as phage display enable us to screen enormous amounts of peptide ligands which fastens drug discovery [151]. However, peptides also have notable drawbacks such as a low plasma stability due to rapid degradation by proteases and a low oral bioavailability [96, 152]. To counteract these limitations, different strategies can be applied to improve the adsorption, distribution, metabolization, and elimination (ADME) properties of peptides such as cyclization, conjugation to macromolecules, replacing l-amino acids with d-isoforms and N-methylation of the peptide bond [153, 154]. To date, over 60 peptide drugs are approved in one of the major markets (USA, EU, and Japan) and over 150 are under development and have entered human clinical trials. A recent overview of all approved (and withdrawn) peptide drugs is given by Lau et al. [96]. Romidepsin is not included in the list as it is a depsipeptide and surprisingly not considered as a peptide drug. Depsipeptides are peptides in which one or more amide bindings are replaced with an esther binding. Nesiritide, the recombinant form of BNP, is approved for the treatment of heart failure. However, its routine use for treatment of chronic heart failure is controversial [97]. Several clinical trials are ongoing to use this peptide in other indications such as diabetes and pulmonary hypertension [155]. No other peptides that target epigenetic mechanisms are yet approved in one of the major markets, and only three others have been or are under clinical development (i.e., lunasin, plitidepsin, and HIP). Recently, a clinical trial using lunasin in the treatment of ALS has been completed. Participants who were receiving lunasin were able to perform slightly better on different functional activities such as swallowing and walking [156, 157]. It was found that H3K9K14ac2 and H4K5K8K12K16 acetylation in blood cells was lower in treated patients compared to untreated controls, demonstrating the inhibitory histone acetylation effects of lunasin. Another clinical trial compared the effect of lunasin with a dietary supplement (Reliv NOW) on cardio-metabolic risk factors, but no results were reported [158]. To date, a dietary supplement containing a high lunasin concentration is already on the market (LunaRich X^TM^, Reliv) and is freely accessible online. Several health claims are being made, and the product is marketed as “the first epigenetic superfood.”

Plitidepsin (Aplidin®), alone or in combination with other treatments, was included in 11 different clinical trials for treating different types of cancer. As already discussed, this peptide was refused market approval by the EMA for the treatment of relapsed/refractory multiple myeloma as its benefits did not outweigh its risks [109].

A first phase 1 clinical trial using HIP for the treatment of diabetes mellitus has been conducted of which no results are available yet [159]. All the other discussed peptides in this review are currently not under clinical development, but are/were investigated in fundamental and/or pre-clinical research.

So, despite the highly potent and promising effects of peptides in modulating epigenetics for the treatment of for example cancer, this does not yet translate in a significant number of therapeutic products which are currently being developed or are on the market. This can be explained by the limitations of peptide drugs such as the low oral availability, the very low plasma stability, and high excretion by the kidneys. Improving the pharmacokinetic properties of peptides by increasing the metabolic stability (i.e., protection against degradation by proteases and peptidases), extending the biological activity (e.g., increased protein binding), increasing membrane association, and changing the tissue distribution and/or excretion rate (e.g., through self-aggregation) can overcome these problems. The pharmacokinetic properties of a peptide are optimized using chemical modifications such as changing l-amino acids with d-amino acids, cyclization, conjugation (e.g., acylation, pegylation, glycosylation), amide bond protection (e.g., N-methyl groups), and incorporation of altered or unnatural amino acids (e.g., tert-butyl group on serin).

An alternative approach for targeting ncRNAs (or mRNA) is the use of peptide nucleic acids (PNAs). These are DNA analogues in which the sugar-phosphate backbone is replaced by N-(2-aminoethyl)glycine units [160]. The main advantage of these PNAs is that they are resistant against both nucleases and proteases and can have a variety of functions such as inhibiting miRNA maturation [161], lncRNA-protein interaction [162], and miRNA-mediated mRNA degradation [163]. While these PNAs are not peptides sensu stricto, they can be linked to cell-penetrating peptides to deliver these active PNAs in the cell where they exert their functions [161, 162, 164].

### Drug repurposing

Drug repurposing or repositioning is the application of known, approved, drugs to new indications which clearly offers cost and time benefits. Different developmental risks are reduced because repositioning candidates have already been through several stages of clinical development and often long-term safety profiles and pharmacokinetic profiles are known [165]. Currently, a lot of research is going on to investigate the epigenetic effects of already FDA-approved drugs and their possible applications in for example cancer and neurodegenerative disorders. By screening an FDA-approved library of 1206 drugs in combination with DNMTi or HDACi, Raynal et al. discovered that 4% (*n* = 45) of the investigated drugs enhanced DNMTi or HDACi activity in a human colon cancer cell line [166]. Hydralazine-valproate is a drug combination being repositioned to an oral DNMT and HDAC inhibitor and is currently in phase II trials for breast cancer and refractory solid tumors and in phase III trials for the treatment of advanced cervical cancer. This combination drug has already been approved in Mexico since 2009 for the treatment of advanced cervical cancer [167]. Chatterjee et al. propose 14 compounds for repositioning in the treatment of Alzheimer’s disease as these drugs share the same epigenetic targets which are involved in AD development [168].

Several approved peptide drugs are already being repositioned to other indications. For example, lisinopril, a synthetic tripeptide derivative approved for treatment of hypertension and heart failure, binds strongly to neuraminidase and has antiviral activity against influenza A [169]. Glatiramer, approved for the treatment of multiple sclerosis, shows promising effects in Huntington’s disease and as an antibiotic [170, 171] and liraglutide against obesity, non-alcoholic fatty liver disease, and depression [172, 173]. Other examples of approved peptide drugs with possible therapeutic effects in other indications exist [174–177]. However, none of the currently approved peptide drugs are under consideration for repositioning due to the discovery of epigenetic effects as none of these peptide drugs, which are under clinical trial for other indications they were initially approved for, have demonstrated epigenetic effects. For the moment, there is an intriguing knowledge gap concerning the possible epigenetic effects of the approved peptide drugs on the market. Seen the potential of peptides to modulate the different epigenetic mechanisms, it is not unlikely that some of the approved peptide drugs exert modulatory epigenetic effects and can have the potential to be used in diseases with a strong epigenetic basis such as cancer or AD [178, 179].

## Conclusion

Today, epigenetic research has attracted pharmaceutical interest. Research is being performed to dissect epigenetic mechanisms in a variety of diseases and to develop small molecule epigenetic drugs. Surprisingly, the therapeutic potential of peptides with epigenetic properties remains underexplored. Despite the many therapeutic advantages of peptides over small molecules, only two (depsi-)peptide drugs with epigenetic modulating properties are currently approved by the FDA and on the market. Drug repurposing can help accelerating marketing approval of already existing peptide drugs for other indications. Currently, none of the existing peptide drugs have been investigated for potential epigenetic effects. Focusing on these potential effects can accelerate therapeutic development of new, highly potent peptides for the treatment of diseases with a strong epigenetic basis such as cancer and/or Alzheimer’s disease.

## Data Availability

Not applicable.

## Abbreviations

AD: Alzheimer’s disease
ADME: Adsorption, distribution, metabolization, and elimination
Aβ: Amyloid-beta
BCM7: Beta-casomorphin-7
BNP: B-type-natriuretic peptide
BRDi: Bromodomain inhibitor
CHAP: Cyclic hydroxamic acid-containing peptide
CTCL: Cutaneous T cell lymphoma
DNMT: DNA methyltransferase
DNMTi: DNA methyltransferase inhibitor
ECJ: European Court of Justice
EMA: European Medicines Agency
ERV: Endogenous retroviruses
HAT: Histone acetyltransferase
HATi: Histone acetyltransferase inhibitor
HDAC: Histone deacetylase
HDACi: Histone deacetylase inhibitor
HDMi: Histone demethylase inhibitor
HEDD: Human epigenetic drug database
HIP: Human pro-islet peptide
HMT: Histone methyltransferase
HMTi: Histone methyltransferase inhibitor
hPTM: Histone post-translational modification
lncRNAs: Long non-coding RNAs
LSD1: Lysine-specific demethylase 1
LTR: Long terminal repeat
MDS: Myelodysplastic syndrome
miRNA: MicroRNA
MM: Multiple myeloma
ncRNAs: Non-coding RNAs
NMD: Nonsense-mediated decay
Nt: Nucleotides
PNAs: Peptide nucleic acids
PTCL: Peripheral T cell lymphoma
RISC: RNA-induced silencing complex
SAM: S-adenosylmethionine
siRNA: Short interfering RNA
SIRTi: Sirtuin inhibitor
TINAT: Treatment-induced non-annotated transcript

## References

[1] Waddington CH (1942). The epigenotype. Endeavour..

[2] Nightingale KP, O'Neill LP, Turner BM (2006). Histone modifications: signalling receptors and potential elements of a heritable epigenetic code. Curr Opin Genet Dev..

[3] Gapp K, Bohacek J (2018). Epigenetic germline inheritance in mammals: looking to the past to understand the future. Genes Brain Behav..

[4] Kazachenka A, Bertozzi TM, Sjoberg-Herrera MK, Walker N, Gardner J, Gunning R (2018). Identification, characterization, and heritability of murine metastable epialleles: implications for non-genetic inheritance. Cell..

[5] Hullar MA, Fu BC (2014). Diet, the gut microbiome, and epigenetics. Cancer J..

[6] Qin Y, Wade PA (2018). Crosstalk between the microbiome and epigenome: messages from bugs. J Biochem..

[7] Abdul QA, Yu BP, Chung HY, Jung HA, Choi JS (2017). Epigenetic modifications of gene expression by lifestyle and environment. Arch Pharm Res..

[8] Cedar H, Bergman Y (2009). Linking DNA methylation and histone modification: patterns and paradigms. Nat Rev Genet.

[9] Tsai M-C, Manor O, Wan Y, Mosammaparast N, Wang JK, Lan F, et al. Long Noncoding RNA as Modular Scaffold of Histone Modification Complexes. Science. 2010;329(5992):689-93. 10.1126/science.1192002 PMC2967777 20616235

[10] Garzon R, Liu S, Fabbri M, Liu Z, Heaphy CE, Callegari E (2009). MicroRNA-29b induces global DNA hypomethylation and tumor suppressor gene reexpression in acute myeloid leukemia by targeting directly DNMT3A and 3B and indirectly DNMT1. Blood..

[11] Prachayasittikul V, Prathipati P, Pratiwi R, Phanus-Umporn C, Malik AA, Schaduangrat N (2017). Exploring the epigenetic drug discovery landscape. Expert Opin Drug Discov..

[12] Cedar H, Bergman Y (2012). Programming of DNA methylation patterns. Annu Rev Biochem..

[13] Deaton AM, Bird A (2011). CpG islands and the regulation of transcription. Genes Dev..

[14] Klutstein M, Nejman D, Greenfield R, Cedar H (2016). DNA Methylation in cancer and aging. Cancer Res..

[15] Kraan CM, Godler DE, Amor DJ. Epigenetics of fragile X syndrome and fragile X-related disorders. Dev Med Child Neurol. 2019;61(2):121-27. 10.1111/dmcn.13985 30084485

[16] Wu SC, Zhang Y (2010). Active DNA demethylation: many roads lead to Rome. Nat Rev Mol Cell Biol..

[17] Reik W, Dean W, Walter J (2001). Epigenetic reprogramming in mammalian development. Science..

[18] Cutter AR, Hayes JJ. A brief review of nucleosome structure. FEBS Lett. 2015;589(20 Pt A):2914-22. 10.1016/j.febslet.2015.05.016 PMC4598263 25980611

[19] Bannister AJ, Kouzarides T (2011). Regulation of chromatin by histone modifications. Cell Res..

[20] Cao LL, Shen C, Zhu WG (2016). Histone modifications in DNA damage response. Sci China Life Sci..

[21] Talbert PB, Henikoff S (2017). Histone variants on the move: substrates for chromatin dynamics. Nat Rev Mol Cell Biol..

[22] Di Ruscio A, Ebralidze AK, Benoukraf T, Amabile G, Goff LA, Terragni J (2013). DNMT1-interacting RNAs block gene-specific DNA methylation. Nature..

[23] Morris KV, Mattick JS (2014). The rise of regulatory RNA. Nat Rev Genet..

[24] Lewis CJ, Pan T, Kalsotra A (2017). RNA modifications and structures cooperate to guide RNA-protein interactions. Nat Rev Mol Cell Biol..

[25] Kadumuri RV, Janga SC (2018). Epitranscriptomic code and its alterations in human disease. Trends Mol Med..

[26] Xiong X, Yi C, Peng J (2017). Epitranscriptomics: toward a better understanding of RNA modifications. Genomics Proteomics Bioinformatics..

[27] Song J, Yi C (2017). Chemical modifications to RNA: a new layer of gene expression regulation. ACS Chem Biol..

[28] Dinescu S, Ignat S, Lazar AD, Constantin C, Neagu M, Costache M. Epitranscriptomic signatures in lncRNAs and their possible roles in cancer. Genes (Basel). 2019;10(1) 10.3390/genes10010052 PMC6356509 30654440

[29] Peer E, Rechavi G, Dominissini D (2017). Epitranscriptomics: regulation of mRNA metabolism through modifications. Curr Opin Chem Biol..

[30] Zhao BS, Roundtree IA, He C (2017). Post-transcriptional gene regulation by mRNA modifications. Nat Rev Mol Cell Biol..

[31] Beermann J, Piccoli MT, Viereck J, Thum T (2016). Non-coding RNAs in development and disease: background, mechanisms, and therapeutic approaches. Physiol Rev..

[32] Wei JW, Huang K, Yang C, Kang CS (2017). Non-coding RNAs as regulators in epigenetics (Review). Oncol Rep..

[33] Du T, Zamore PD (2005). microPrimer: the biogenesis and function of microRNA. Development..

[34] Bartel DP (2009). MicroRNAs: target recognition and regulatory functions. Cell..

[35] Mohr AM, Mott JL (2015). Overview of microRNA biology. Semin Liver Dis..

[36] Kozomara A, Griffiths-Jones S (2014). miRBase: annotating high confidence microRNAs using deep sequencing data. Nucleic Acids Res..

[37] Kaikkonen MU, Lam MT, Glass CK (2011). Non-coding RNAs as regulators of gene expression and epigenetics. Cardiovasc Res..

[38] Ruiz-Orera J, Messeguer X, Subirana JA, Alba MM (2014). Long non-coding RNAs as a source of new peptides. Elife..

[39] Pang Y, Mao C, Liu S (2018). Encoding activities of non-coding RNAs. Theranostics..

[40] Wyck S, Herrera C, Requena CE, Bittner L, Hajkova P, Bollwein H (2018). Oxidative stress in sperm affects the epigenetic reprogramming in early embryonic development. Epigenetics Chromatin..

[41] Pan W, Yu H, Zheng B, Gao Y, Li P, Huang Q (2017). Upregulation of MiR-369-3p suppresses cell migration and proliferation by targeting SOX4 in Hirschsprung’s disease. J Pediatr Surg..

[42] Wang G, Zhang L, Wang H, Cui M, Liu W, Liu Y (2018). Demethylation of GFRA4 promotes cell proliferation and invasion in Hirschsprung disease. DNA Cell Biol..

[43] Bennett RL, Licht JD (2018). Targeting epigenetics in cancer. Annu Rev Pharmacol Toxicol..

[44] Shi L, Cui S, Engel JD, Tanabe O (2013). Lysine-specific demethylase 1 is a therapeutic target for fetal hemoglobin induction. Nat Med..

[45] Cacabelos R, Torrellas C, Teijido O, Carril JC (2016). Pharmacogenetic considerations in the treatment of Alzheimer’s disease. Pharmacogenomics..

[46] Pavlou MAS, Pinho R, Paiva I, Outeiro TF (2017). The yin and yang of alpha-synuclein-associated epigenetics in Parkinson’s disease. Brain..

[47] Gandal MJ, Zhang P, Hadjimichael E, Walker RL, Chen C, Liu S, et al. Transcriptome-wide isoform-level dysregulation in ASD, schizophrenia, and bipolar disorder. Science. 2018;362(6420):eaat8127. 10.1126/science.aat8127 PMC6443102 30545856

[48] Salta E, De Strooper B (2017). Noncoding RNAs in neurodegeneration. Nat Rev Neurosci..

[49] Conickx G, Mestdagh P, Avila Cobos F, Verhamme FM, Maes T, Vanaudenaerde BM (2017). MicroRNA profiling reveals a role for microRNA-218-5p in the pathogenesis of chronic obstructive pulmonary disease. Am J Respir Crit Care Med..

[50] De Smet EG, Mestdagh P, Vandesompele J, Brusselle GG, Bracke KR (2015). Non-coding RNAs in the pathogenesis of COPD. Thorax..

[51] Berdasco M, Esteller M (2019). Clinical epigenetics: seizing opportunities for translation. Nat Rev Genet..

[52] Hibner G, Kimsa-Furdzik M, Francuz T. Relevance of microRNAs as potential diagnostic and prognostic markers in colorectal cancer. Int J Mol Sci. 2018;19(10) 10.3390/ijms19102944 PMC6213499 30262723

[53] Li X, Zhao Z, Gao C, Rao L, Hao P, Jian D (2018). Identification of a peripheral blood long non-coding RNA (Upperhand) as a potential diagnostic marker of coronary artery disease. Cardiol J..

[54] Nagaraj S, Zoltowska KM, Laskowska-Kaszub K, Wojda U. microRNA diagnostic panel for Alzheimer’s disease and epigenetic trade-off between neurodegeneration and cancer. Ageing Res Rev. 2019;49:125-43. 10.1016/j.arr.2018.10.008 30391753

[55] Ashapkin VV, Linkova NS, Khavinson V, Vanyushin BF (2014). Epigenetic mechanisms of peptidergic regulation of gene expression during aging of human cells. Biochemistry (Mosc)..

[56] Barucker C, Sommer A, Beckmann G, Eravci M, Harmeier A, Schipke CG (2015). Alzheimer amyloid peptide abeta42 regulates gene expression of transcription and growth factors. J Alzheimers Dis..

[57] Khavinson V, Tendler SM, Vanyushin BF, Kasyanenko NA, Kvetnoy IM, Lin'kova NS (2014). Peptide regulation of gene expression and protein synthesis in bronchial epithelium. Lung..

[58] Shan ZG, Zhu KX, Chen FY, Liu J, Chen B, Qiao K (2016). In vivo activity and the transcriptional regulatory mechanism of the antimicrobial peptide SpHyastatin in Scylla paramamosain. Fish Shellfish Immunol..

[59] Khavinson VK, Lin'kova NS, Tarnovskaya SI (2016). Short peptides regulate gene expression. Bull Exp Biol Med..

[60] Khavinson VK, Solov'ev AY, Tarnovskaya SI, Lin'kova NS (2013). Mechanism of biological activity of short peptides: cell penetration and epigenetic regulation of gene expression. Biology Bulletin Reviews..

[61] Khavinson V, Solov'ev A, Zhilinskii DV, Shataeva LK, Vaniushin BF (2012). Epigenetic aspects of peptide regulation of aging. Adv Gerontol..

[62] Chen KL, Wang SS, Yang YY, Yuan RY, Chen RM, Hu CJ (2009). The epigenetic effects of amyloid-beta(1-40) on global DNA and neprilysin genes in murine cerebral endothelial cells. Biochem Biophys Res Commun..

[63] Hodgson N, Trivedi M, Muratore C, Li S, Deth R (2013). Soluble oligomers of amyloid-beta cause changes in redox state, DNA methylation, and gene transcription by inhibiting EAAT3 mediated cysteine uptake. J Alzheimers Dis..

[64] Obeid R, Hubner U, Bodis M, Geisel J (2016). Plasma amyloid beta 1-42 and DNA methylation pattern predict accelerated aging in young subjects with Down syndrome. Neuromolecular Med..

[65] Trivedi MS, Hodgson NW, Walker SJ, Trooskens G, Nair V, Deth RC (2015). Epigenetic effects of casein-derived opioid peptides in SH-SY5Y human neuroblastoma cells. Nutr Metab (Lond).

[66] Trivedi MS, Shah JS, Al-Mughairy S, Hodgson NW, Simms B, Trooskens GA (2014). Food-derived opioid peptides inhibit cysteine uptake with redox and epigenetic consequences. J Nutr Biochem..

[67] Trivedi M, Zhang Y, Lopez-Toledano M, Clarke A, Deth R (2016). Differential neurogenic effects of casein-derived opioid peptides on neuronal stem cells: implications for redox-based epigenetic changes. J Nutr Biochem..

[68] Jiang Z, Shi D, Tu Y, Tian J, Zhang W, Xing B (2018). Human proislet peptide promotes pancreatic progenitor cells to ameliorate diabetes through FOXO1/menin-mediated epigenetic regulation. Diabetes..

[69] Smadbeck J, Peterson MB, Zee BM, Garapaty S, Mago A, Lee C (2014). De novo peptide design and experimental validation of histone methyltransferase inhibitors. PLoS One..

[70] Morrison MJ, Boriack-Sjodin PA, Swinger KK, Wigle TJ, Sadalge D, Kuntz KW (2018). Identification of a peptide inhibitor for the histone methyltransferase WHSC1. PLoS One..

[71] Kumarasinghe IR, Woster PM (2018). Cyclic peptide inhibitors of lysine-specific demethylase 1 with improved potency identified by alanine scanning mutagenesis. Eur J Med Chem..

[72] Jeong HJ, Jeong JB, Kim DS, de Lumen BO (2007). Inhibition of core histone acetylation by the cancer preventive peptide lunasin. J Agric Food Chem..

[73] Harrison SJ, Bishton M, Bates SE, Grant S, Piekarz RL, Johnstone RW (2012). A focus on the preclinical development and clinical status of the histone deacetylase inhibitor, romidepsin (depsipeptide, Istodax((R))). Epigenomics..

[74] Biggins JB, Gleber CD, Brady SF (2011). Acyldepsipeptide HDAC inhibitor production induced in Burkholderia thailandensis. Org Lett..

[75] Klausmeyer P, Shipley SM, Zuck KM, McCloud TG (2011). Histone deacetylase inhibitors from Burkholderia thailandensis. J Nat Prod..

[76] Wang C, Henkes LM, Doughty LB, He M, Wang D, Meyer-Almes FJ (2011). Thailandepsins: bacterial products with potent histone deacetylase inhibitory activities and broad-spectrum antiproliferative activities. J Nat Prod..

[77] Chen Y, Gambs C, Abe Y, Wentworth P, Janda KD (2003). Total synthesis of the depsipeptide FR-901375. J Org Chem..

[78] Hong J, Luesch H (2012). Largazole: from discovery to broad-spectrum therapy. Nat Prod Rep..

[79] Alonso-Alvarez S, Pardal E, Sanchez-Nieto D, Navarro M, Caballero MD, Mateos MV (2017). Plitidepsin: design, development, and potential place in therapy. Drug Des Devel Ther..

[80] De Schepper S, Hln B, Verhulst T, Steller U, Andries L, Wouters W (2003). Inhibition of histone deacetylases by chlamydocin induces apoptosis and proteasome-mediated degradation of survivin. J Pharmacol Exp Ther..

[81] Porter NJ, Christianson DW (2017). Binding of the microbial cyclic tetrapeptide trapoxin A to the class I histone deacetylase HDAC8. ACS Chem Biol..

[82] Furumai R, Komatsu Y, Nishino N, Khochbin S, Yoshida M, Horinouchi S (2001). Potent histone deacetylase inhibitors built from trichostatin A and cyclic tetrapeptide antibiotics including trapoxin. Proc Natl Acad Sci U S A..

[83] Komatsu Y, Tomizaki KY, Tsukamoto M, Kato T, Nishino N, Sato S (2001). Cyclic hydroxamic-acid-containing peptide 31, a potent synthetic histone deacetylase inhibitor with antitumor activity. Cancer Res..

[84] Ahn MY, Ahn SG, Yoon JH (2011). Apicidin, a histone deaceylase inhibitor, induces both apoptosis and autophagy in human oral squamous carcinoma cells. Oral Oncol..

[85] Gu W, Cueto M, Jensen PR, Fenical W, Silverman RB. Microsporins A and B: new histone deacetylase inhibitors from the marine-derived fungus Microsporum cf. gypseum and the solid-phase synthesis of microsporin A. Tetrahedron. 2007;63(28):6535-6541.

[86] Nakao Y, Yoshida S, Matsunaga S, Shindoh N, Terada Y, Nagai K (2006). Azumamides A-E: histone deacetylase inhibitory cyclic tetrapeptides from the marine sponge Mycale izuensis. Angewandte Chemie International Edition..

[87] Mori H, Urano Y, Abe F, Furukawa S, Tsurumi Y, Sakamoto K (2003). FR235222, a fungal metabolite, is a novel immunosuppressant that inhibits mammalian histone deacetylase (HDAC). I. Taxonomy, fermentation, isolation and biological activities. J Antibiot (Tokyo).

[88] Sasamura S, Sakamoto K, Takagaki S, Yamada T, Takase S, Mori H (2010). AS1387392, a novel immunosuppressive cyclic tetrapeptide compound with inhibitory activity against mammalian histone deacetylase. J Antibiot (Tokyo)..

[89] Kim S, Lee JH, Kang I, Hyun S, Yu J, Shin C (2016). An amphiphilic peptide induces apoptosis through the miR29b-p53 pathway in cancer cells. Mol Ther Nucleic Acids..

[90] Pai J, Hyun S, Hyun JY, Park SH, Kim WJ, Bae SH (2016). Screening of pre-miRNA-155 binding peptides for apoptosis inducing activity using peptide microarrays. J Am Chem Soc..

[91] Shortridge MD, Walker MJ, Pavelitz T, Chen Y, Yang W, Varani G (2017). A macrocyclic peptide ligand binds the oncogenic microRNA-21 precursor and suppresses dicer processing. ACS Chem Biol..

[92] Zhang X, Sha M, Yao Y, Da J, Jing D (2015). Increased B-type-natriuretic peptide promotes myocardial cell apoptosis via the B-type-natriuretic peptide/long non-coding RNA LSINCT5/caspase-1/interleukin 1beta signaling pathway. Mol Med Rep..

[93] Lin'kova NS, Drobintseva AO, Orlova OA, Kuznetsova EP, Polyakova VO, Kvetnoy IM (2016). Peptide regulation of skin fibroblast functions during their aging in vitro. Bull Exp Biol Med..

[94] Ishino M, Takeishi Y, Niizeki T, Watanabe T, Nitobe J, Miyamoto T (2008). Risk stratification of chronic heart failure patients by multiple biomarkers: implications of BNP, H-FABP, and PTX3. Circ J..

[95] Sackner-Bernstein JD, Kowalski M, Fox M, Aaronson K (2005). Short-term risk of death after treatment with nesiritide for decompensated heart failure: a pooled analysis of randomized controlled trials. JAMA..

[96] Lau JL, Dunn MK (2018). Therapeutic peptides: historical perspectives, current development trends, and future directions. Bioorg Med Chem..

[97] O'Connor CM, Starling RC, Hernandez AF, Armstrong PW, Dickstein K, Hasselblad V (2011). Effect of nesiritide in patients with acute decompensated heart failure. N Engl J Med..

[98] Wan X, Liu H, Sun Y, Zhang J, Chen X, Chen N (2017). Lunasin: a promising polypeptide for the prevention and treatment of cancer. Oncol Lett..

[99] Liu J, Jia SH, Kirberger M, Chen N (2014). Lunasin as a promising health-beneficial peptide. Eur Rev Med Pharmacol Sci..

[100] Sarkar A, Gogia N, Glenn N, Singh A, Jones G, Powers N (2018). A soy protein Lunasin can ameliorate amyloid-beta 42 mediated neurodegeneration in Drosophila eye. Sci Rep..

[101] Group TA (2014). ALSUntangled No. 26: lunasin. Amyotroph Lateral Scler Frontotemporal Degener..

[102] Wu YC, Liu X, Wang JL, Chen XL, Lei L, Han J (2016). Soft-shelled turtle peptide modulates microRNA profile in human gastric cancer AGS cells. Oncol Lett..

[103] Wynendaele E, De Spiegeleer B, Gevaert B, Janssens Y, Suleman S, Cattoor S (2018). Regulatory status of N-alkylamide containing health products. Regul Toxicol Pharmacol..

[104] Fulbright NR. Borderline between foods and drugs. http://curia.europa.eu/juris/liste.jsf?language=nl&num=C-140/07. Accessed 10 Jan 2019.

[105] Taevernier L, Wynendaele E, Gevaert B, De Spiegeleer B (2017). Chemical classification of cyclic depsipeptides. Curr Protein Pept Sci..

[106] Qi Y, Wang D, Jin T, Yang L, Wu H, Li Y, et al. HEDD: the human epigenetic drug database. Database (Oxford). 2016:2016. 10.1093/database/baw159 PMC5199199 28025347

[107] Leisch M, Egle A, Greil R. Plitidepsin: a potential new treatment for relapsed/refractory multiple myeloma. Future Oncol. 2018. 10.2217/fon-2018-0492 30111169

[108] Mateos M-V, Cibeira MT, Richardson P, Blade J, Prosper F, Oriol A (2008). Final results of a phase II trial with plitidepsin (Aplidin) alone and in combination with dexamethasone in patients with relapsed/refractory multiple myeloma. Blood.

[109] EMA. Refusal of the marketing authorisation for Aplidin (plitidepsin). https://ema.europa.eu/en/medicines/human/EPAR/aplidin. Accessed on 10/12/2018.

[110] Du L, Risinger AL, King JB, Powell DR, Cichewicz RH (2014). A potent HDAC inhibitor, 1-alaninechlamydocin, from a Tolypocladium sp. induces G2/M cell cycle arrest and apoptosis in MIA PaCa-2 cells. J Nat Prod..

[111] Bhuiyan MP, Kato T, Okauchi T, Nishino N, Maeda S, Nishino TG (2006). Chlamydocin analogs bearing carbonyl group as possible ligand toward zinc atom in histone deacetylases. Bioorg Med Chem..

[112] Nishino N, Jose B, Shinta R, Kato T, Komatsu Y, Yoshida M (2004). Chlamydocin-hydroxamic acid analogues as histone deacetylase inhibitors. Bioorg Med Chem..

[113] Wang S, Li X, Wei Y, Xiu Z, Nishino N (2014). Discovery of potent HDAC inhibitors based on chlamydocin with inhibitory effects on cell migration. ChemMedChem..

[114] Hoque MA, Islam MS, Islam MN, Kato T, Nishino N, Ito A (2014). Design and synthesis of mono and bicyclic tetrapeptides thioester as potent inhibitor of histone deacetylases. Amino Acids..

[115] Montero A, Beierle JM, Olsen CA, Ghadiri MR (2009). Design, synthesis, biological evaluation, and structural characterization of potent histone deacetylase inhibitors based on cyclic alpha/beta-tetrapeptide architectures. J Am Chem Soc..

[116] Forneris F, Binda C, Adamo A, Battaglioli E, Mattevi A (2007). Structural basis of LSD1-CoREST selectivity in histone H3 recognition. J Biol Chem..

[117] Amano Y, Kikuchi M, Sato S, Yokoyama S, Umehara T, Umezawa N (2017). Development and crystallographic evaluation of histone H3 peptide with N-terminal serine substitution as a potent inhibitor of lysine-specific demethylase 1. Bioorg Med Chem..

[118] Kakizawa T, Ota Y, Itoh Y, Suzuki T (2018). Histone H3 peptides incorporating modified lysine residues as lysine-specific demethylase 1 inhibitors. Bioorg Med Chem Lett..

[119] Ota Y, Kakizawa T, Itoh Y, Suzuki T. Design, synthesis, and in vitro evaluation of novel histone H3 peptide-based LSD1 inactivators incorporating α,α-disubstituted amino acids with γ-turn-inducing structures. Molecules. 2018;23(5):E1099. 10.3390/molecules23051099 PMC6099693 29734782

[120] Itoh Y, Aihara K, Mellini P, Tojo T, Ota Y, Tsumoto H (2016). Identification of SNAIL1 peptide-based irreversible lysine-specific demethylase 1-selective inactivators. J Med Chem..

[121] Simon JA, Lange CA (2008). Roles of the EZH2 histone methyltransferase in cancer epigenetics. Mutat Res..

[122] Qi W, Chan H, Teng L, Li L, Chuai S, Zhang R (2012). Selective inhibition of Ezh2 by a small molecule inhibitor blocks tumor cells proliferation. Proc Natl Acad Sci U S A..

[123] Bose D, Nahar S, Rai MK, Ray A, Chakraborty K, Maiti S (2015). Selective inhibition of miR-21 by phage display screened peptide. Nucleic Acids Res..

[124] Sakamoto K, Otake K, Umemoto T (2017). Discovery of peptidic miR-21 processing inhibitor by mirror image phage display: a novel method to generate RNA binding D-peptides. Bioorg Med Chem Lett..

[125] Glahn A, Rhein M, Frieling H, Schuster R, El Aissami A, Bleich S (2017). Smoking and promoter-specific deoxyribonucleic acid methylation of the atrial natriuretic peptide gene: methylation of smokers and non-smokers differs significantly during withdrawal. Eur Addict Res..

[126] Glahn A, Rhein M, Heberlein A, Muschler M, Kornhuber J, Frieling H (2016). The epigenetic regulation of GATA4-dependent brain natriuretic peptide expression during alcohol withdrawal. Neuropsychobiology..

[127] Glahn A, Riera Knorrenschild R, Rhein M, Haschemi Nassab M, Groschl M, Heberlein A (2017). Alcohol-induced changes in methylation status of individual CpG sites, and serum levels of vasopressin and atrial natriuretic peptide in alcohol-dependent patients during detoxification treatment. Eur Addict Res..

[128] Hillemacher T, Frieling H, Luber K, Yazici A, Muschler MA, Lenz B (2009). Epigenetic regulation and gene expression of vasopressin and atrial natriuretic peptide in alcohol withdrawal. Psychoneuroendocrinology..

[129] Yedery RD, Jerse AE (2015). Augmentation of cationic antimicrobial peptide production with histone deacetylase inhibitors as a novel epigenetic therapy for bacterial infections. Antibiotics (Basel)..

[130] Philippeit C, Busch M, Dunker N (2014). Epigenetic control of trefoil factor family (TFF) peptide expression in human retinoblastoma cell lines. Cell Physiol Biochem..

[131] Perry JK, Kannan N, Grandison PM, Mitchell MD, Lobie PE (2008). Are trefoil factors oncogenic?. Trends Endocrinol Metab..

[132] Brocks D, Schmidt CR, Daskalakis M, Jang HS, Shah NM, Li D (2017). DNMT and HDAC inhibitors induce cryptic transcription start sites encoded in long terminal repeats. Nat Genet..

[133] Licht JD (2015). DNA methylation inhibitors in cancer therapy: the immunity dimension. Cell..

[134] Chiappinelli KB, Strissel PL, Desrichard A, Li H, Henke C, Akman B (2015). Inhibiting DNA methylation causes an interferon response in cancer via dsRNA including endogenous retroviruses. Cell..

[135] Li J, Hao D, Wang L, Wang H, Wang Y, Zhao Z (2017). Epigenetic targeting drugs potentiate chemotherapeutic effects in solid tumor therapy. Sci Rep..

[136] Mazzone R, Zwergel C, Mai A, Valente S (2017). Epi-drugs in combination with immunotherapy: a new avenue to improve anticancer efficacy. Clin Epigenetics..

[137] Suraweera A, O'Byrne KJ, Richard DJ (2018). Combination therapy with histone deacetylase inhibitors (HDACi) for the treatment of cancer: achieving the full therapeutic potential of HDACi. Front Oncol..

[138] Conforti F, Davies ER, Calderwood CJ, Thatcher TH, Jones MG, Smart DE (2017). The histone deacetylase inhibitor, romidepsin, as a potential treatment for pulmonary fibrosis. Oncotarget..

[139] Hui KF, Cheung AK, Choi CK, Yeung PL, Middeldorp JM, Lung ML (2016). Inhibition of class I histone deacetylases by romidepsin potently induces Epstein-Barr virus lytic cycle and mediates enhanced cell death with ganciclovir. Int J Cancer..

[140] Nebbioso A, Carafa V, Benedetti R, Altucci L (2012). Trials with ‘epigenetic’ drugs: an update. Mol Oncol..

[141] Gottlicher M, Minucci S, Zhu P, Kramer OH, Schimpf A, Giavara S (2001). Valproic acid defines a novel class of HDAC inhibitors inducing differentiation of transformed cells. EMBO J..

[142] Lee BH, Yegnasubramanian S, Lin X, Nelson WG (2005). Procainamide is a specific inhibitor of DNA methyltransferase 1. J Biol Chem..

[143] Zeisberg E, Zeisberg M. A rationale for epigenetic repurposing of hydralazine in chronic heart and kidney failure. J Clin Epigenet. 2016;2(1). 10.21767/2472-1158.100011.

[144] Laribi K, Alani M, Truong C (2018). Baugier de Materre A. Recent advances in the treatment of peripheral T-cell lymphoma. Oncologist..

[145] Kataria BC, Mehta DS, Chhaiya SB (2013). Drug lag for cardiovascular drug approvals in India compared with the US and EU approvals. Indian Heart J..

[146] EMA. Istodax. https://www.ema.europa.eu/en/medicines/human/EPAR/istodax. Accessed on 14/06/2019.

[147] Monga V, Swami U, Tanas M, Bossler A, Mott SL, Smith BJ, et al. A phase I/II study targeting angiogenesis using bevacizumab combined with chemotherapy and a histone deacetylase inhibitor (valproic acid) in advanced sarcomas. Cancers (Basel). 2018;10(2):E53. 10.3390/cancers10020053 PMC5836085 29462961

[148] Lv L, Ge W, Liu Y, Lai G, Liu H, Li W (2016). Lysine-specific demethylase 1 inhibitor rescues the osteogenic ability of mesenchymal stem cells under osteoporotic conditions by modulating H3K4 methylation. Bone Res..

[149] Lu X, Ning Z, Li Z, Cao H, Wang X (2016). Development of chidamide for peripheral T-cell lymphoma, the first orphan drug approved in China. Intractable Rare Dis Res..

[150] Global Peptide Therapeutics Market, Dosage, Price & Clinical Trials Insight 2024. https://www.researchandmarkets.com/reports/4600544/global-peptide-therapeutics-market-dosage-price. Accessed on 18/06/2019.

[151] Saw PE, Song EW. Phage display screening of therapeutic peptide for cancer targeting and therapy. Protein Cell. 2019. 10.1007/s13238-019-0639-7 10.1007/s13238-019-0639-7 PMC6834755 31140150

[152] Fosgerau K, Hoffmann T (2018). Peptide therapeutics: current status and future directions. Drug Discov Today..

[153] Di L (2015). Strategic approaches to optimizing peptide ADME properties. AAPS J..

[154] Rader AFB, Reichart F, Weinmuller M, Kessler H (2018). Improving oral bioavailability of cyclic peptides by N-methylation. Bioorg Med Chem..

[155] Effect of nesiritide infusion on insulin sensitivity in healthy obese insulin resistant subjects (BNP3). https://clinicaltrials.gov/. Accessed on 15/01/2019.

[156] Rooney J, Burke T, Vajda A, Heverin M, Hardiman O (2017). What does the ALSFRS-R really measure? A longitudinal and survival analysis of functional dimension subscores in amyotrophic lateral sclerosis. J Neurol Neurosurg Psychiatry..

[157] Bedlack R. ALS Reversals - Lunasin Regimen. https://clinicaltrials.gov/. Accessed on 17/12/2018.

[158] Sabate J. Effects of soy based dietary supplements on cardiometabolic risk factors. https://clinicaltrials.gov/. Accessed on 17/12/2018.

[159] A study of subcutaneous doses of HIP2B in healthy male subjects (HIP2B). https://clinicaltrials.gov/. Accessed on 15/01/2019.

[160] Fabbri E, Brognara E, Borgatti M, Lampronti I, Finotti A, Bianchi N (2011). miRNA therapeutics: delivery and biological activity of peptide nucleic acids targeting miRNAs. Epigenomics..

[161] Avitabile C, Saviano M, D'Andrea L, Bianchi N, Fabbri E, Brognara E (2012). Targeting pre-miRNA by peptide nucleic acids: a new strategy to interfere in the miRNA maturation. Artif DNA PNA XNA..

[162] Ozes AR, Wang Y, Zong X, Fang F, Pilrose J, Nephew KP (2017). Therapeutic targeting using tumor specific peptides inhibits long non-coding RNA HOTAIR activity in ovarian and breast cancer. Sci Rep..

[163] Zarrilli F, Amato F, Morgillo CM, Pinto B, Santarpia G, Borbone N, et al. Peptide nucleic acids as miRNA target protectors for the treatment of cystic fibrosis. Molecules. 2017;22(7):E1144. 10.3390/molecules22071144 PMC6152032 28698463

[164] Cohen JL, Shen Y, Aouadi M, Vangala P, Tencerova M, Amano SU (2016). Peptide- and amine-modified glucan particles for the delivery of therapeutic siRNA. Mol Pharm..

[165] Ashburn TT, Thor KB (2004). Drug repositioning: identifying and developing new uses for existing drugs. Nat Rev Drug Discov..

[166] Raynal NJ, Da Costa EM, Lee JT, Gharibyan V, Ahmed S, Zhang H (2017). Repositioning FDA-approved drugs in combination with epigenetic drugs to reprogram colon cancer epigenome. Mol Cancer Ther..

[167] Duenas-Gonzalez A, Coronel J, Cetina L, Gonzalez-Fierro A, Chavez-Blanco A, Taja-Chayeb L (2014). Hydralazine-valproate: a repositioned drug combination for the epigenetic therapy of cancer. Expert Opin Drug Metab Toxicol..

[168] Chatterjee P, Roy D, Rathi N (2018). Epigenetic drug repositioning for Alzheimer’s disease based on epigenetic targets in human interactome. J Alzheimers Dis..

[169] Rohini K, Shanthi V (2018). Hyphenated 3D-QSAR statistical model-drug repurposing analysis for the identification of potent neuraminidase inhibitor. Cell Biochem Biophys..

[170] Christiansen SH, Murphy RA, Juul-Madsen K, Fredborg M, Hvam ML, Axelgaard E (2017). The immunomodulatory drug glatiramer acetate is also an effective antimicrobial agent that kills gram-negative bacteria. Sci Rep..

[171] Corey-Bloom J, Jia H, Aikin AM, Thomas EA (2014). Disease modifying potential of glatiramer acetate in Huntington’s disease. J Huntingtons Dis..

[172] Moreira GV, Azevedo FF, Ribeiro LM, Santos A, Guadagnini D, Gama P (2018). Liraglutide modulates gut microbiota and reduces NAFLD in obese mice. J Nutr Biochem..

[173] Weina H, Yuhu N, Christian H, Birong L, Feiyu S, Le W (1694). Liraglutide attenuates the depressive- and anxiety-like behaviour in the corticosterone induced depression model via improving hippocampal neural plasticity. Brain Res..

[174] Zhu H, Stern RA, Tao Q, Bourlas A, Essis MD, Chivukula M (2017). An amylin analog used as a challenge test for Alzheimer’s disease. Alzheimers Dement (N Y)..

[175] Aviles-Olmos I, Dickson J, Kefalopoulou Z, Djamshidian A, Ell P, Soderlund T (2013). Exenatide and the treatment of patients with Parkinson’s disease. J Clin Invest..

[176] Appleby BS, Cummings JL (2013). Discovering new treatments for Alzheimer’s disease by repurposing approved medications. Curr Top Med Chem..

[177] Soave CL, Guerin T, Liu J, Dou QP (2017). Targeting the ubiquitin-proteasome system for cancer treatment: discovering novel inhibitors from nature and drug repurposing. Cancer Metastasis Rev..

[178] Dehghani R, Rahmani F, Rezaei N (2018). MicroRNA in Alzheimer’s disease revisited: implications for major neuropathological mechanisms. Rev Neurosci..

[179] Lord J, Cruchaga C (2014). The epigenetic landscape of Alzheimer's disease. Nat Neurosci..

